# Supplementary material for: Macromolecules Absorbed from Influenza Infection-Based Sera Modulate the Cellular Uptake of Polymeric Nanoparticles
Source: Biomimetics (Basel). 2022 Nov 30;7(4):219. doi: 10.3390/biomimetics7040219 (PMC9775140; doi:10.3390/biomimetics7040219)
Supplement: Supplementary file 1 [file biomimetics-07-00219-s001.zip › biomimetics-2029717-supplementary.pdf]

Macromolecules absorbed from influenza infection-based sera modulate the cellular uptake of polymeric nanoparticles

Daniel Nierenberg[1], Orielyz Flores[1], David Fox[2,3], Yuen Yee Li Sip[2,4], Caroline Finn[1], Heba Ghozlan[1], Amanda Cox[1], Melanie Coathup[5], K. Kai McKinstry[1], Lei Zhai[2,3,4], and Annette R Khaled[1]*

[1]Burnett School of Biomedical Sciences, College of Medicine, University of Central Florida, Orlando, FL, USA; [2]Nanotechnology Science Center, University of Central Florida, Orlando, FL, USA; [3]Department of Chemistry, College of Science, University of Central Florida, Orlando, FL, USA; [4]Department of Materials Science and Engineering, College of Engineering and Computer Science, University of Central Florida, Orlando, FL, USA; [5]Biionix Cluster and Department of Medicine, College of Medicine, University of Central Florida, Orlando, FL, USA

*Corresponding author

Corresponding author email address: Annette.Khaled@ucf.edu

Content:

Total number of pages: 8

Total number of figures: 6

SUPPORTING INFORMATION

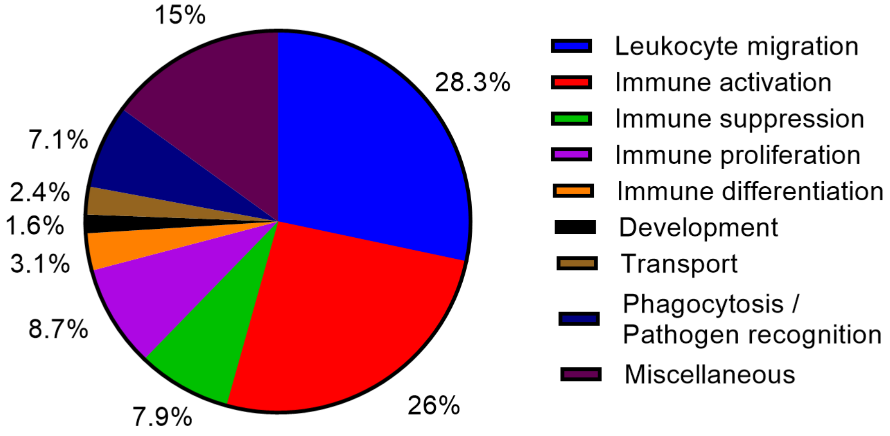

Figure S1. Immune-related sheddome

Known or predicted shed proteins related to the immune system, organized by category. Frequency of proteins (n = 127) within each category are represented as a percentage. Proteins were identified through SheddomeDB or DeepSMP (A Deep Learning Model for Predicting the Shedding Events of Membrane Proteins) databases.

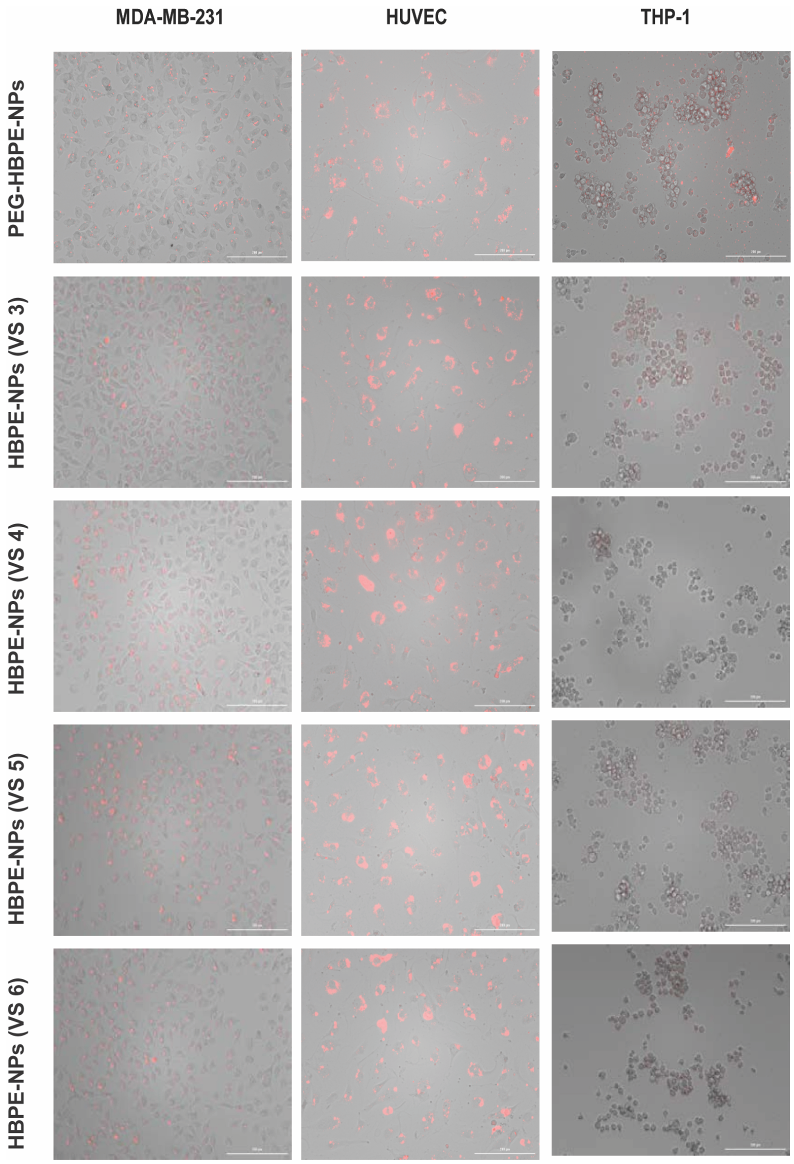

Figure S2. HBPE-NPs pre-coated with sera collected from mice infected with IAV display improved cancer cell uptake and reduced monocyte uptake over PEG-HBPE-NPs

Uptake of HBPE-NPs coated with influenza A virus (IAV)-infected mouse sera (days 3 to 6 of infection) was compared to uptake of PEG-HBPE-NPs. Representative Cytation 5 microscopy images 24 h post-treatment of MDA-MB-231 (left column), HUVEC (middle column), and THP-1 (right column) cells with DiI dye-encapsulated PEG-HBPE-NPs (topmost row), HBPE-

NPs (VS3) (second row), HBPE-NPs (VS4) (third row), HBPE-NPs (VS5) (fourth row), and HBPE-NPs (VS6) (fifth row). Red fluorescence depicts nanoparticle uptake in cells (DiI dye presence). Scale bar represents 200 µm. Magnification was at 10X.

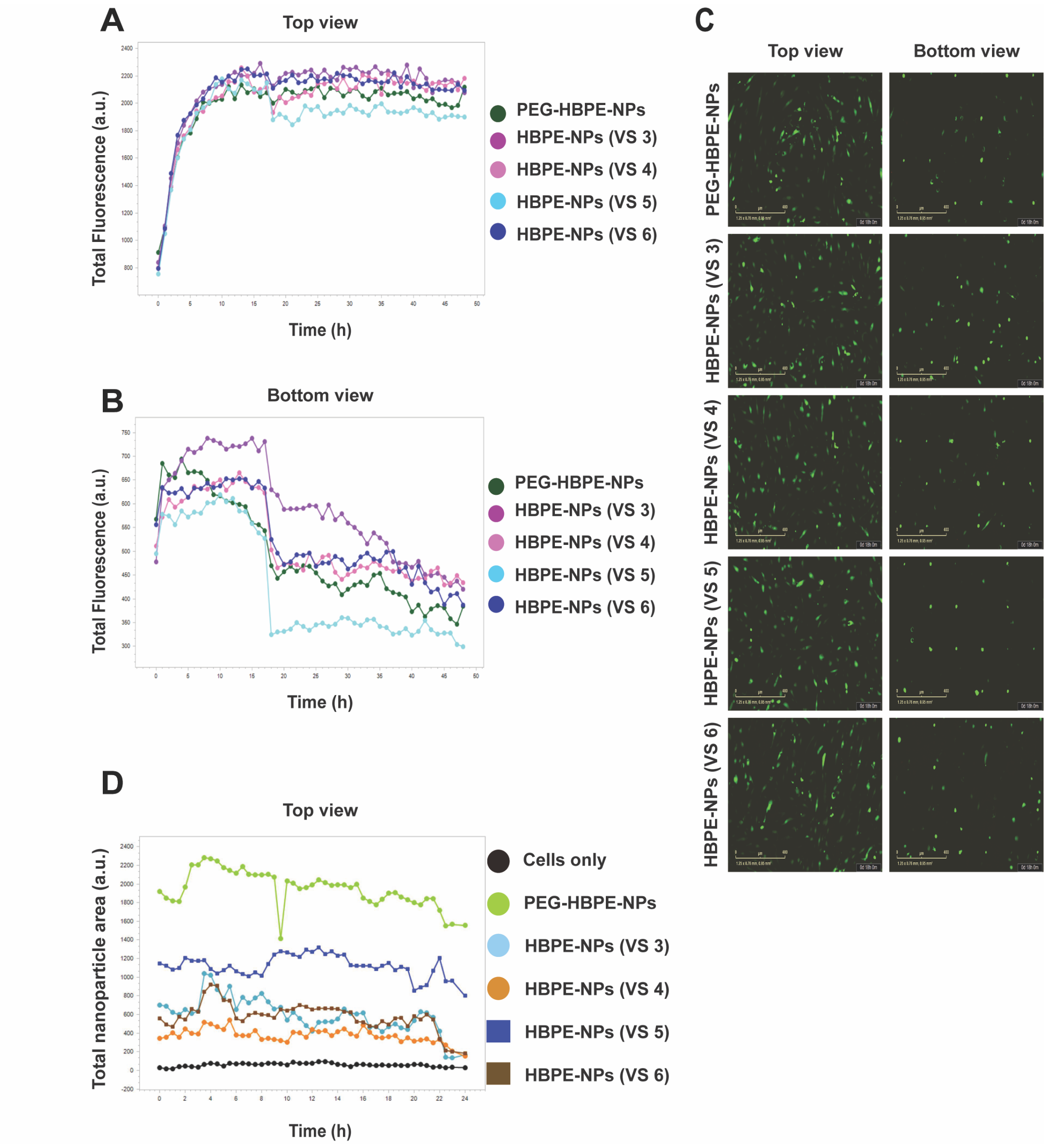

Figure S3. HBPE-NPs pre-coated with sera collected from IAV-infected mice do not promote the migration of endothelial cells.

Employing the Incucyte Live-Cell analysis system, HUVEC cells (Cytolight Green) total green fluorescence graphs are shown (A) above or (B) below pore filters for 48 hours (1-hour increments) of treatment with DiI-encapsulated PEG-HBPE-NPs, HBPE- NP (VS3) HBPE-NP (VS3), HBPE-NP (VS4), HBPE-NP (VS5), and HBPE-NP (VS6). (C) Representative HUVEC fluorescent images at 18 hours post-treatment with PEG-HBPE-NPs, HBPE-NPs (VS3), HBPE-NPs (VS4), HBPE-NPs (VS5), and HBPE-NPs (VS6). Magnification was at 10X. (D) Incucyte Live-Cell analysis system was used to quantify total red fluorescence count signal of DiI-loaded PEG-HBPE-NPs, HBPE-NPs (VS3), HBPE-NPs (VS4), HBPE-NPs (VS5), HBPE-NPs (VS6) after 24 hours (30 min increments) of treatment with GFP-HUVEC cells. Data represents mean total RFP (HBPE-NPs) or GFP (HUVECs) count per well.

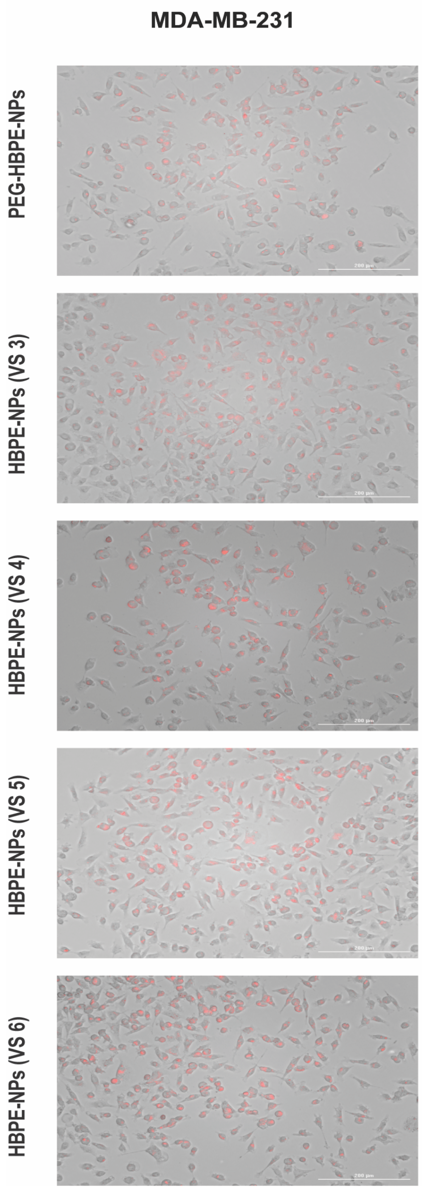

Figure S4. HBPE-NPs pre-coated with sera collected from IAV-infected mice reveal improved migration and cancer cell uptake

Uptake of HBPE-NPs that were coated with sera from influenza A virus (IAV)-infected mouse sera (days 3 to 6 of infection) was compared to PEG-HBPE-NPs. Representative Cytation 5 microscopy images were taken 24 hour post-treatment of HUVECs (top chamber) with DiI dye-encapsulated HBPE-NPs and uptake visualized by imaging MDA-MB-231 cells (bottom

chamber). Panels are PEG-HBPE-NPs (topmost panel), HBPE-NPs (VS3) (second panel), HBPE-NPs (VS4) (third panel), HBPE-NPs (VS5) (fourth panel), and HBPE-NPs (VS6) (fifth panel). Red fluorescence depicts nanoparticle uptake in cells (DiI dye presence). Scale bar represents 200 μm. Magnification was at 10X.

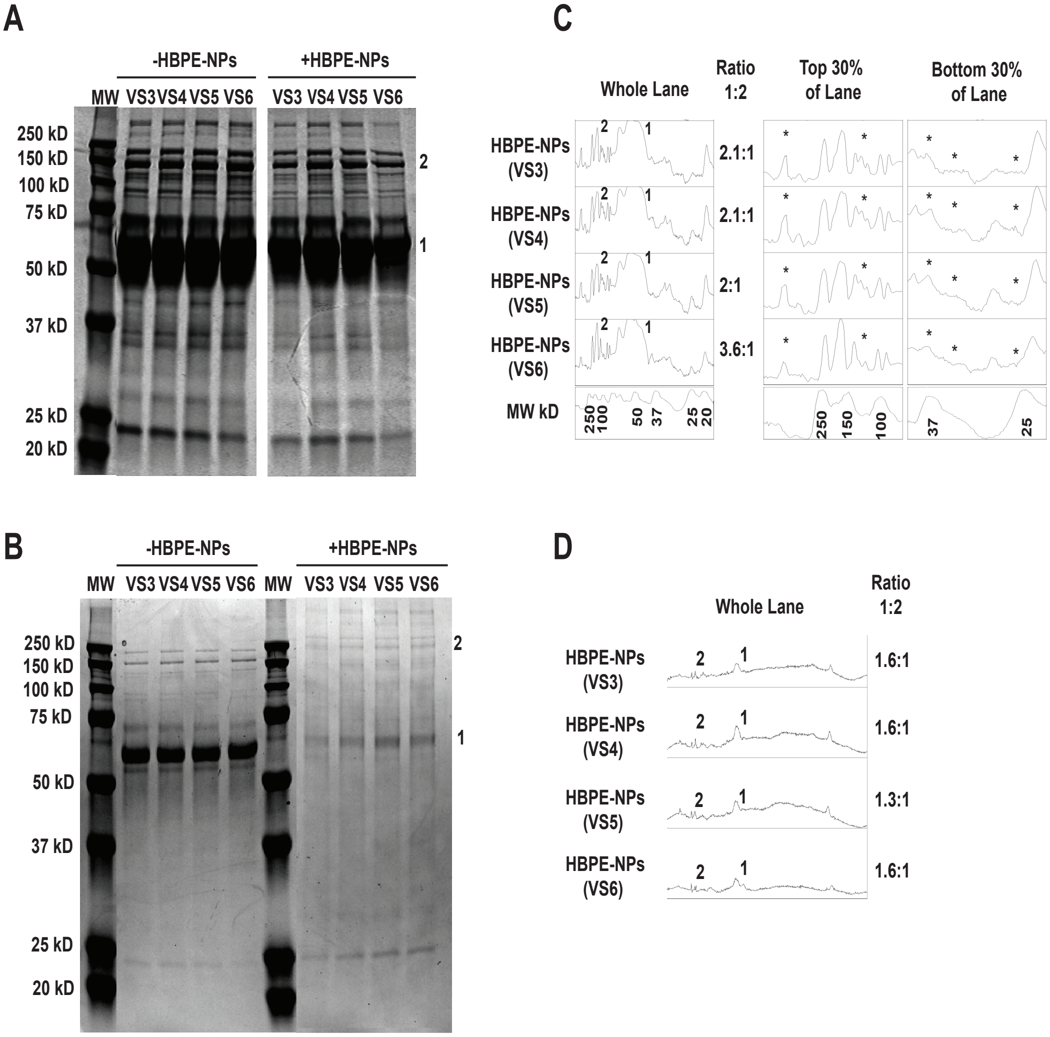

Figure S5. Unique profiles result from proteins absorbed by HBPE-NPs pre-treated with sera collected from mice at different stages of the IAV infection.

Proteins in VS3-6 without NPs (-HBPE-NPs) and proteins absorbed by HBPE-NPs treated with VS3-6 (+HBPE-NPs) were assessed by SDS-PAGE and visualized by Coomassie staining. HBPE-NPs were treated with 80 μg VS3-6 (A, C) or 20 μg VS3-6 (B, D). A Precision-Plus Protein Dual Color protein reference ladder was used for MW comparison. Histograms of individual lane patterns are shown for the whole lane, the upper third of lane, or the lower third of a lane for 80 μg sera (C) and 20 μg sera (D). Two peaks (1, 2) were selected for quantitation, and ratios of peaks 1 to 2 are shown. Histograms were created and quantified using ImageJ gel analysis software.

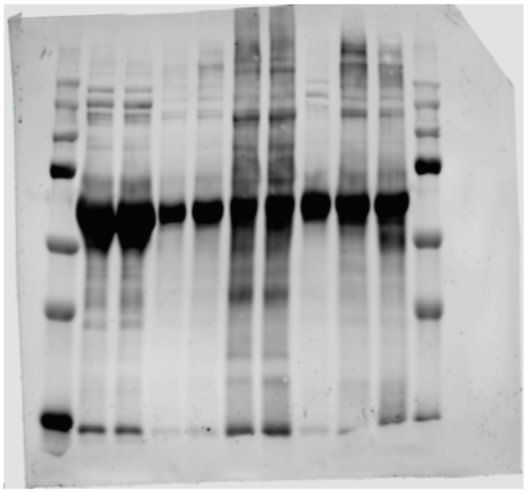

Figure S6. Raw data blot for Figure 6.

List of mass spectrometry data containing identified coronal proteins on HBPE-NPs

Abbreviations:

IAV	Influenza A virus
HBPE-NP	Carboxylated-Hyperbranched polyester-nanoparticle
V3 (w)	Quantitative Value (Normalized Total Spectra) of whole IAV-infected sera 3 days post-infection
V5 (w)	Quantitative Value (Normalized Total Spectra) of whole IAV-infected sera 5 days post-infection
V3 (c)	Quantitative Value (Normalized Total Spectra) of IAV-infected sera 3 days post-infection processed the same as V3 + HBPE-NP
V5 (c)	Quantitative Value (Normalized Total Spectra) of IAV-infected sera 5 days post-infection processed the same as V5 + HBPE-NP
V3 + HBPE-NP	Quantitative Value (Normalized Total Spectra) of COOH-HBPE-NP protein corona after incubation with IAV-infected sera 3 days post-infection
V5 + HBPE-NP	Quantitative Value (Normalized Total Spectra) of COOH-HBPE-NP protein corona after incubation with IAV-infected sera 5 days post-infection

Proteins listed by descending order of Quantitative Value (Normalized Total Spectra) for V5 + NP

**Protein name**	**Accession Number**	**Molecular Weight**	**V3 (w)**	**V5 (w)**	**V3 (c)**	**V5 (c)**	**V3 + HBPE-NP**	**V5 + HBPE-NP**
Albumin	ALBU_MOUSE	69 kDa	971.26	828.18	619.34	541.9	455.45	530.96
Cluster of Pregnancy zone protein	PZP_MOUSE [2]	166 kDa	146.13	152.88	104.36	76.489	139.9	219.86
Pregnancy zone protein	PZP_MOUSE	166 kDa	146.13	152.88	104.36	76.489	139.9	219.86
Cluster of Alpha-1-antitrypsin 1-4	A1AT4_MOUSE [4]	46 kDa	86.702	74.789	53.313	55.746	261.56	217.13
Cluster of GLOBIN domain-containing protein	A8DUK4_MOUSE [3]	16 kDa	148.08	89.087	66.925	58.339	223.54	213.48
GLOBIN domain-containing protein	A8DUK4_MOUSE	16 kDa	148.08	89.087	66.925	58.339	222.78	212.56
Complement C3	CO3_MOUSE	186 kDa	163.66	174.88	153.13	140.01	233.43	186.11
Alpha-1-antitrypsin 1-4	A1AT4_MOUSE	46 kDa	66.244	53.892	38.567	44.078	188.57	153.27
Alpha-1-antitrypsin 1-1	A1AT1_MOUSE	46 kDa	48.709	40.694	32.895	31.114	187.04	149.62
Alpha-1-antitrypsin 1-2	A1AT2_MOUSE	46 kDa	53.58	42.894	31.761	31.114	168.8	145.05
Serotransferrin	TRFE_MOUSE	77 kDa	312.71	238.67	198.5	160.76	133.06	122.25
Hemopexin	HEMO_MOUSE	51 kDa	103.26	104.49	61.253	80.378	103.41	113.12
Cluster of Serine protease inhibitor A3K	SPA3K_MOUSE [5]	47 kDa	74.038	64.891	49.91	45.375	107.97	112.21
Cluster of Murinoglobulin-1	MUG1_MOUSE [3]	165 kDa	62.348	56.092	85.074	67.414	130.02	108.56
Murinoglobulin-1	MUG1_MOUSE	165 kDa	62.348	56.092	85.074	67.414	130.02	108.56
Alpha-1-antitrypsin 1-5	A1AT5_MOUSE	46 kDa	22.406	15.398	10.209	9.0749	130.78	93.966
Serine protease inhibitor A3K	SPA3K_MOUSE	47 kDa	60.399	53.892	36.298	37.596	79.076	82.106
Fibronectin	FINC_MOUSE	273 kDa	45.787	56.092	73.73	75.192	58.546	76.633
Plasminogen	PLMN_MOUSE	91 kDa	70.141	94.587	74.865	55.746	72.233	66.597
Complement C4-B	CO4B_MOUSE	193 kDa	22.406	18.697	27.224	23.336	63.108	62.036
Inter alpha-trypsin inhibitor, heavy chain 4	ITIH4_MOUSE	105 kDa	30.2	45.094	89.611	89.453	79.836	61.124
Apolipoprotein A-I	APOA1_MOUSE	31 kDa	70.141	73.69	116.83	155.57	63.869	59.299
Hemoglobin subunit alpha	HBA_MOUSE	15 kDa	70.141	38.495	46.507	47.967	82.877	56.562
Apolipoprotein B-100	APOB_MOUSE	509 kDa	9.7418	20.897	6.8059	27.225	36.496	53.825
Hemoglobin subunit beta-2	HBB2_MOUSE	16 kDa	57.477	32.995	24.955	18.15	49.422	52.913
Complement factor H	CFAH_MOUSE	139 kDa	55.528	70.39	56.716	59.635	31.174	51.088
Kininogen-1	KNG1_MOUSE	73 kDa	54.554	50.593	89.611	82.971	54.745	49.264
Serine protease inhibitor A3M	SPA3M_MOUSE	47 kDa	32.148	34.095	23.821	23.336	41.059	47.439
Vitamin D-binding protein	VTDB_MOUSE	54 kDa	37.993	34.095	38.567	46.671	49.422	47.439
Haptoglobin	HPT_MOUSE	39 kDa	43.838	48.393	38.567	47.967	17.488	47.439
Murinoglobulin-2	MUG2_MOUSE	162 kDa	37.019	29.696	46.507	40.189	50.943	39.229
Hemoglobin subunit epsilon-Y2	HBE_MOUSE	16 kDa	0	0	0	0	29.653	36.492
Cluster of Carboxylesterase 1C	EST1C_MOUSE [3]	61 kDa	49.683	34.095	27.224	20.743	35.736	36.492
Apolipoprotein A-IV	APOA4_MOUSE	45 kDa	20.458	38.495	38.567	46.671	36.496	33.755
Carboxylesterase 1C	EST1C_MOUSE	61 kDa	47.735	31.896	27.224	20.743	33.455	33.755
Serine protease inhibitor A3N	SPA3N_MOUSE	47 kDa	22.406	23.097	15.88	15.557	30.414	31.018
Prothrombin	THRB_MOUSE	70 kDa	24.355	31.896	38.567	44.078	33.455	29.193
Phosphatidylinositol-glycan-specific phospholipase D	PHLD_MOUSE	93 kDa	16.561	18.697	22.686	31.114	33.455	28.281
Inter-alpha-trypsin inhibitor heavy chain H3	ITIH3_MOUSE	99 kDa	9.7418	19.797	20.418	23.336	25.091	26.457
Inter-alpha-trypsin inhibitor heavy chain H1	ITIH1_MOUSE	101 kDa	3.8967	7.6989	6.8059	6.4821	27.372	26.457
HMW kininogen-II	Q6S9I3_MOUSE	71 kDa	12.664	13.198	20.418	18.15	23.571	25.544
Thrombospondin-1	TSP1_MOUSE	130 kDa	23.38	37.395	71.462	62.228	26.612	22.807
Inter-alpha-trypsin inhibitor heavy chain H2	ITIH2_MOUSE	106 kDa	9.7418	18.697	21.552	24.632	28.893	22.807
Ceruloplasmin	CERU_MOUSE	121 kDa	53.58	58.292	27.224	32.41	6.8431	21.895
Beta-2-glycoprotein 1	APOH_MOUSE	39 kDa	30.2	27.496	23.821	33.707	7.6034	21.895
Complement factor B	CFAB_MOUSE	85 kDa	20.458	24.197	22.686	23.336	28.133	21.895
Antithrombin-III	ANT3_MOUSE	52 kDa	14.613	15.398	14.746	11.668	25.091	21.895
Inhibitor of carbonic anhydrase	ICA_MOUSE	77 kDa	19.484	25.296	11.343	10.371	24.331	19.158
Histidine-rich glycoprotein	HRG_MOUSE	59 kDa	15.587	19.797	11.343	19.446	22.81	19.158
Cluster of Actin, cytoplasmic 1	ACTB_MOUSE [5]	42 kDa	5.8451	9.8986	14.746	10.371	18.248	19.158
Cluster of IF rod domain-containing protein	E9Q0F0_MOUSE [18]	112 kDa	12.664	18.697	11.343	35.003	3.8017	18.246
Alpha-2-HS-glycoprotein	FETUA_MOUSE	37 kDa	43.838	38.495	81.671	75.192	31.174	17.334
Cluster of H-2 class I histocompatibility antigen, Q10 alpha chain	HA10_MOUSE [8]	37 kDa	9.7418	9.8986	26.089	42.782	22.05	17.334
H-2 class I histocompatibility antigen, Q10 alpha chain	HA10_MOUSE	37 kDa	8.7676	8.7988	23.821	38.893	19.769	17.334
Complement factor I	CFAI_MOUSE	67 kDa	11.69	14.298	18.149	23.336	17.488	17.334
BPI fold-containing family A member 2	BPIA2_MOUSE	25 kDa	1.9484	6.5991	9.0745	22.039	6.0827	17.334
Clusterin	CLUS_MOUSE	52 kDa	18.509	16.498	49.91	47.967	25.091	16.421
Gelsolin	GELS_MOUSE	86 kDa	12.664	18.697	35.164	33.707	32.695	16.421
Actin, cytoplasmic 1	ACTB_MOUSE (+1)	42 kDa	4.8709	9.8986	12.477	10.371	16.728	16.421
Carboxylic ester hydrolase	D3Z5G7_MOUSE	62 kDa	21.432	16.498	12.477	11.668	12.926	15.509
Vitronectin	VTNC_MOUSE	55 kDa	7.7935	12.098	41.97	33.707	27.372	15.509
Plasma kallikrein	KLKB1_MOUSE	71 kDa	9.7418	8.7988	9.0745	11.668	17.488	15.509
Carboxypeptidase N subunit 2	CPN2_MOUSE	60 kDa	3.8967	8.7988	9.0745	5.1857	18.248	14.597
Serum paraoxonase/arylesterase 1	PON1_MOUSE	40 kDa	6.8193	12.098	9.0745	9.0749	11.405	14.597
Alpha-1B-glycoprotein	A1BG_MOUSE	57 kDa	2.9225	8.7988	4.5373	11.668	9.8845	14.597
Immunoglobulin heavy constant mu	IGHM_MOUSE	50 kDa	24.355	29.696	19.283	19.446	6.0827	13.684
Zinc-alpha-2-glycoprotein	ZA2G_MOUSE	35 kDa	6.8193	7.6989	7.9402	6.4821	12.926	13.684
Complement C5	CO5_MOUSE	189 kDa	13.639	16.498	9.0745	6.4821	20.529	12.772
Apolipoprotein E	APOE_MOUSE	36 kDa	10.716	12.098	17.015	23.336	11.405	12.772
Plasma protease C1 inhibitor	IC1_MOUSE	56 kDa	7.7935	7.6989	6.8059	3.8893	15.967	12.772
Serine protease inhibitor A3C	SPA3C_MOUSE	47 kDa	15.587	20.897	11.343	16.853	12.926	11.86
Serine protease inhibitor A3F	SPA3F_MOUSE	50 kDa	0	0	0	0	0	11.86
Alpha-1-acid glycoprotein 1	A1AG1_MOUSE	24 kDa	4.8709	3.2995	3.4029	1.2964	15.207	11.86
Fetuin-B	FETUB_MOUSE	43 kDa	14.613	15.398	22.686	25.928	12.926	10.948
Alpha-2-antiplasmin	A2AP_MOUSE	55 kDa	7.7935	12.098	11.343	5.1857	13.686	10.948
Cluster of Glutathione peroxidase 3	GPX3_MOUSE [2]	25 kDa	8.7676	14.298	4.5373	11.668	3.8017	10.948
Glutathione peroxidase 3	GPX3_MOUSE	25 kDa	8.7676	14.298	4.5373	11.668	3.8017	10.948
Insulin-like growth factor-binding protein complex acid labile subunit	ALS_MOUSE	67 kDa	1.9484	1.0998	0	0	6.0827	10.948
Afamin	AFAM_MOUSE	69 kDa	5.8451	3.2995	5.6716	3.8893	20.529	10.035
Complement component C8 beta chain	CO8B_MOUSE	66 kDa	5.8451	7.6989	11.343	11.668	7.6034	10.035
Sulfhydryl oxidase 1	QSOX1_MOUSE	83 kDa	8.7676	9.8986	4.5373	5.1857	3.0414	10.035
Actin, alpha skeletal muscle	ACTS_MOUSE	42 kDa	4.8709	6.5991	10.209	7.7785	9.8845	9.1229
Protein AMBP	AMBP_MOUSE	39 kDa	13.639	18.697	22.686	24.632	6.8431	9.1229
Ig gamma-2B chain C region	IGG2B_MOUSE	44 kDa	9.7418	21.997	3.4029	23.336	0.76034	9.1229
Epidermal growth factor receptor	EGFR_MOUSE	135 kDa	5.8451	9.8986	11.343	10.371	10.645	9.1229
Complement component C8 alpha chain	CO8A_MOUSE	66 kDa	2.9225	4.3994	5.6716	7.7785	12.165	9.1229
Immunoglobulin heavy constant gamma 3	A0A1Y7VJN6_MOUSE (+1)	44 kDa	4.8709	4.3994	1.1343	5.1857	0	8.2106
Ig gamma-1 chain C region, membrane-bound form	IGH1M_MOUSE	43 kDa	16.561	25.296	11.343	28.521	0.76034	7.2984
Coagulation factor X	FA10_MOUSE	54 kDa	8.7676	8.7988	11.343	12.964	7.6034	7.2984
LRRCT domain-containing protein	Q91XL1_MOUSE	37 kDa	9.7418	7.6989	6.8059	5.1857	6.8431	7.2984
Serum amyloid A-1 protein	SAA1_MOUSE	14 kDa	9.7418	6.5991	21.552	18.15	10.645	7.2984
Apolipoprotein C-III	APOC3_MOUSE	11 kDa	0	0	4.5373	19.446	5.3224	7.2984
Complement component C8 gamma chain	CO8G_MOUSE	23 kDa	0.97418	3.2995	0	0	2.281	7.2984
Alpha-1-acid glycoprotein 2	A1AG2_MOUSE	24 kDa	8.7676	7.6989	5.6716	1.2964	6.0827	7.2984

Keratin, type II cytoskeletal 1	K2C1_MOUSE	66 kDa	3.8967	7.6989	4.5373	12.964	1.5207	6.3861
Keratin, type II cytoskeletal 1b	K2C1B_MOUSE	61 kDa	3.8967	3.2995	2.2686	5.1857	2.281	6.3861
Immunoglobulin kappa constant	IGKC_MOUSE	12 kDa	9.7418	18.697	12.477	23.336	2.281	6.3861
Apolipoprotein A-II	APOA2_MOUSE	11 kDa	5.8451	4.3994	5.6716	7.7785	15.207	6.3861
Corticosteroid-binding globulin	CBG_MOUSE	45 kDa	5.8451	5.4992	5.6716	1.2964	8.3638	6.3861
Complement factor D	CFAD_MOUSE	28 kDa	2.9225	3.2995	5.6716	3.8893	8.3638	6.3861
Phosphatidylcholine-sterol acyltransferase	LCAT_MOUSE	50 kDa	0.97418	0	1.1343	1.2964	6.0827	6.3861
C4b-binding protein	C4BPA_MOUSE	52 kDa	2.9225	4.3994	4.5373	6.4821	5.3224	5.4738
Coagulation factor XII	FA12_MOUSE	66 kDa	0.97418	2.1997	3.4029	3.8893	8.3638	5.4738
Protein Z-dependent protease inhibitor	ZPI_MOUSE	52 kDa	0.97418	1.0998	1.1343	0	3.8017	5.4738
Retinol-binding protein 4	RET4_MOUSE	23 kDa	0	0	2.2686	1.2964	3.8017	5.4738
Ig-like domain-containing protein	Q8HWB2_MOUSE	40 kDa	2.9225	4.3994	9.0745	16.853	6.0827	4.5615
H-2 class I histocompatibility antigen, Q8 alpha chain	HA18_MOUSE	37 kDa	1.9484	2.1997	0	5.1857	0	4.5615
Ig-like domain-containing protein	G3UXE9_MOUSE	45 kDa	0	0	0	0	0	4.5615
Beta-actin-like protein 2	ACTBL_MOUSE	42 kDa	2.9225	3.2995	7.9402	3.8893	3.0414	4.5615
Cluster of Keratin, type I cytoskeletal 10	K1C10_MOUSE [9]	58 kDa	5.8451	14.298	6.8059	25.928	0	4.5615
Complement component C9	CO9_MOUSE	62 kDa	4.8709	1.0998	5.6716	1.2964	10.645	4.5615
Vitamin K-dependent protein Z	PROZ_MOUSE	44 kDa	3.8967	4.3994	5.6716	5.1857	3.8017	4.5615
Ig-like domain-containing protein	A0A075B5M7_MOUSE	10 kDa	4.8709	8.7988	2.2686	6.4821	0	4.5615
IF rod domain-containing protein	E9Q0F0_MOUSE	112 kDa	3.8967	4.3994	3.4029	6.4821	0.76034	3.6492
Keratin, type II cytoskeletal 73	K2C73_MOUSE	59 kDa	2.9225	2.1997	2.2686	5.1857	1.5207	3.6492
Keratin, type II cytoskeletal 74	K2C74_MOUSE	55 kDa	2.9225	0	0	0	1.5207	3.6492
Beta-2-microglobulin	B2MG_MOUSE	14 kDa	2.9225	2.1997	13.612	12.964	2.281	3.6492
Serum amyloid A-4 protein	SAA4_MOUSE	15 kDa	2.9225	2.1997	4.5373	3.8893	9.1241	3.6492
Cluster of Talin-1	TLN1_MOUSE [2]	270 kDa	0	0	7.9402	6.4821	3.0414	3.6492
Talin-1	TLN1_MOUSE	270 kDa	0	0	7.9402	6.4821	3.0414	3.6492
Lumican	LUM_MOUSE	38 kDa	1.9484	4.3994	6.8059	6.4821	3.0414	3.6492
Mannose-binding protein C	MBL2_MOUSE	26 kDa	0.97418	3.2995	6.8059	6.4821	2.281	3.6492
Heparin cofactor 2	HEP2_MOUSE	54 kDa	1.9484	3.2995	2.2686	2.5928	1.5207	3.6492
Ras-related protein Rab-21	RAB21_MOUSE	24 kDa	3.8967	4.3994	2.2686	5.1857	3.0414	3.6492
Coagulation factor XIII B chain	F13B_MOUSE	76 kDa	1.9484	0	4.5373	1.2964	3.0414	3.6492
N-acetylmuramoyl-L-alanine amidase	PGRP2_MOUSE	58 kDa	0.97418	4.3994	2.2686	2.5928	4.5621	3.6492
Carbonic anhydrase 1	CAH1_MOUSE	28 kDa	1.9484	1.0998	0	1.2964	4.5621	3.6492
Carboxylesterase 1D	EST1D_MOUSE	62 kDa	6.8193	3.2995	3.4029	0	2.281	2.7369
Keratin, type II cytoskeletal 2 epidermal	K22E_MOUSE	71 kDa	1.9484	3.2995	0	5.1857	0	2.7369
Keratin, type I cytoskeletal 10	K1C10_MOUSE	58 kDa	5.8451	12.098	4.5373	10.371	0	2.7369
Apolipoprotein C-I	APOC1_MOUSE	10 kDa	0	0	11.343	11.668	2.281	2.7369
Talin-2	TLN2_MOUSE	254 kDa	0	0	1.1343	0	0	2.7369
Carboxypeptidase N catalytic chain	CBPN_MOUSE	52 kDa	4.8709	6.5991	4.5373	3.8893	3.8017	2.7369
Hepatocyte growth factor activator	HGFA_MOUSE	71 kDa	4.8709	8.7988	5.6716	6.4821	3.0414	2.7369
Serum amyloid A-2 protein	SAA2_MOUSE	14 kDa	4.8709	1.0998	21.552	15.557	6.8431	2.7369
Serum amyloid P-component	SAMP_MOUSE	26 kDa	5.8451	8.7988	4.5373	3.8893	0.76034	2.7369
Carbonic anhydrase 2	CAH2_MOUSE	29 kDa	5.8451	2.1997	1.1343	0	6.8431	2.7369
Coagulation factor V	FA5_MOUSE	247 kDa	0	1.0998	6.8059	3.8893	3.8017	2.7369
Cluster of Ig-like domain-containing protein	A0A075B5V1_MOUSE [3]	11 kDa	2.9225	7.6989	3.4029	7.7785	0	2.7369
Macrophage colony-stimulating factor 1 receptor	CSF1R_MOUSE	109 kDa	1.9484	3.2995	1.1343	0	4.5621	2.7369
Cluster of Complement C1s-A subcomponent	CS1A_MOUSE [2]	77 kDa	0	0	0	0	5.3224	2.7369
Complement C1s-A subcomponent	CS1A_MOUSE	77 kDa	0	0	0	0	5.3224	2.7369
Complement C1s-B subcomponent	CS1B_MOUSE	77 kDa	0	0	0	0	2.281	2.7369
Carboxypeptidase B2	CBPB2_MOUSE	49 kDa	0	0	0	0	2.281	2.7369
Ig-like domain-containing protein	A0A0A6YWI9_MOUSE	13 kDa	0	2.1997	0	0	0	2.7369
Cluster of Tubulin beta-1 chain	TBB1_MOUSE [5]	50 kDa	0	0	1.1343	0	0	2.7369
Tubulin beta-1 chain	TBB1_MOUSE	50 kDa	0	0	0	0	0	2.7369
Ig-like domain-containing protein	A0A0G2JE99_MOUSE (+1)	12 kDa	2.9225	1.0998	1.1343	1.2964	0	2.7369
Ig kappa chain V-V region HP R16.7	KV5AB_MOUSE (+2)	12 kDa	0	0	0	0	0	2.7369
Keratin, type II cytoskeletal 5	K2C5_MOUSE	62 kDa	0.97418	2.1997	2.2686	2.5928	0.76034	1.8246
Keratin, type II cytoskeletal 2 oral	K22O_MOUSE	63 kDa	0.97418	0	1.1343	3.8893	0	1.8246
IF rod domain-containing protein	E9Q1Z0_MOUSE	58 kDa	0	0	0	2.5928	0.76034	1.8246
Keratin, type II cytoskeletal 79	K2C79_MOUSE	58 kDa	0	0	0	2.5928	0	1.8246
Keratin, type I cytoskeletal 42	K1C42_MOUSE	50 kDa	0	2.1997	2.2686	11.668	0	1.8246
Keratin, type I cytoskeletal 15	K1C15_MOUSE	49 kDa	2.9225	7.6989	2.2686	7.7785	0	1.8246
Keratin, type I cytoskeletal 14	K1C14_MOUSE	53 kDa	2.9225	7.6989	2.2686	10.371	0	1.8246
Transthyretin	TTHY_MOUSE	16 kDa	11.69	9.8986	7.9402	7.7785	1.5207	1.8246
Immunoglobulin heavy constant gamma 2C	F6TQW2_MOUSE	44 kDa	5.8451	12.098	1.1343	3.8893	0	1.8246
Golgi autoantigen, golgin subfamily b, macrogolgin 1	E9PVZ8_MOUSE	370 kDa	0	0	0	0	0.76034	1.8246
Cluster of Maltase-glucoamylase	A0A571BF69_MOUSE [2]	413 kDa	1.9484	1.0998	0	0	6.0827	1.8246
Maltase-glucoamylase	A0A571BF69_MOUSE	413 kDa	1.9484	1.0998	0	0	6.0827	1.8246
Peroxiredoxin-2	PRDX2_MOUSE	22 kDa	0.97418	2.1997	4.5373	2.5928	3.8017	1.8246
Platelet-activating factor acetylhydrolase	PAFA_MOUSE	49 kDa	0	3.2995	6.8059	3.8893	0.76034	1.8246
Ig-like domain-containing protein	A0A075B5V1_MOUSE	11 kDa	2.9225	7.6989	3.4029	7.7785	0	1.8246
IGv domain-containing protein	A0A075B5U4_MOUSE	11 kDa	0	1.0998	0	0	0	1.8246
Apolipoprotein C-IV	APOC4_MOUSE	14 kDa	2.9225	1.0998	4.5373	6.4821	3.0414	1.8246
Flavin reductase (NADPH)	BLVRB_MOUSE	22 kDa	0.97418	1.0998	1.1343	1.2964	5.3224	1.8246
Adiponectin	ADIPO_MOUSE	27 kDa	1.9484	3.2995	2.2686	1.2964	0.76034	1.8246
Cluster of Ig-like domain-containing protein	A0A075B5R5_MOUSE [4]	13 kDa	2.9225	3.2995	1.1343	3.8893	0.76034	1.8246
Ig-like domain-containing protein	A0A075B5R5_MOUSE	13 kDa	2.9225	3.2995	1.1343	3.8893	0.76034	1.8246
Coagulation factor VII	FA7_MOUSE	50 kDa	0	1.0998	3.4029	2.5928	1.5207	1.8246
Mannan-binding lectin serine protease 1	MASP1_MOUSE	80 kDa	0	2.1997	0	0	2.281	1.8246
VWFA domain-containing protein	E9Q7P1_MOUSE	160 kDa	0	2.1997	1.1343	1.2964	1.5207	1.8246
Vitamin K-dependent protein S	PROS_MOUSE	75 kDa	0	0	3.4029	2.5928	2.281	1.8246
Ig-like domain-containing protein	A0A140T8M5_MOUSE	13 kDa	0.97418	3.2995	0	3.8893	0	1.8246
Tubulin alpha-4A chain	TBA4A_MOUSE	50 kDa	0	0	0	0	3.8017	1.8246
Ig-like domain-containing protein	A0A0B4J1N0_MOUSE	11 kDa	0	2.1997	0	0	0	1.8246
Ig-like domain-containing protein	A0A0A6YYE7_MOUSE	13 kDa	0	0	0	0	0	1.8246
Keratin, type I cytoskeletal 16	K1C16_MOUSE	52 kDa	2.9225	6.5991	1.1343	7.7785	0	0.91229
Apolipoprotein D	APOD_MOUSE	22 kDa	1.9484	5.4992	15.88	28.521	1.5207	0.91229
THO complex subunit 5 homolog	THOC5_MOUSE	79 kDa	2.9225	1.0998	0	1.2964	0.76034	0.91229
Apolipoprotein M	APOM_MOUSE	21 kDa	3.8967	3.2995	3.4029	1.2964	0.76034	0.91229
Nesprin-2	SYNE2_MOUSE	783 kDa	0	0	0	0	0.76034	0.91229
Centromere-associated protein E	CENPE_MOUSE	287 kDa	0	0	0	0	0	0.91229
Apolipoprotein N	G3X9D6_MOUSE	28 kDa	1.9484	2.1997	0	0	3.0414	0.91229
Secreted phosphoprotein 24	SPP24_MOUSE	23 kDa	1.9484	1.0998	4.5373	7.7785	2.281	0.91229
Complement C1r-A subcomponent	C1RA_MOUSE	80 kDa	0.97418	2.1997	2.2686	1.2964	2.281	0.91229
Ig-like domain-containing protein	A0A075B5V7_MOUSE	11 kDa	0.97418	2.1997	1.1343	1.2964	0	0.91229
Phospholipid transfer protein	PLTP_MOUSE	54 kDa	0	0	2.2686	1.2964	2.281	0.91229
Interleukin-1 receptor accessory protein	IL1AP_MOUSE	66 kDa	1.9484	3.2995	2.2686	1.2964	1.5207	0.91229
HECT and RLD domain-containing E3 ubiquitin protein ligase family member	E9PZP8_MOUSE	533 kDa	0.97418	2.1997	1.1343	2.5928	0.76034	0.91229
Cluster of Glyceraldehyde-3-phosphate dehydrogenase	G3P_MOUSE [2]	36 kDa	0	2.1997	4.5373	6.4821	1.5207	0.91229
Glyceraldehyde-3-phosphate dehydrogenase	G3P_MOUSE	36 kDa	0	2.1997	4.5373	6.4821	1.5207	0.91229
Vitamin K-dependent protein C	PROC_MOUSE	52 kDa	0	1.0998	5.6716	5.1857	1.5207	0.91229
Ig lambda-2 chain C region	LAC2_MOUSE	11 kDa	1.9484	7.6989	1.1343	6.4821	0	0.91229
Cluster of Ig-like domain-containing protein	A0A075B5T2_MOUSE [3]	13 kDa	4.8709	6.5991	3.4029	1.2964	0	0.91229
Ig-like domain-containing protein	A0A075B5T2_MOUSE	13 kDa	4.8709	6.5991	3.4029	1.2964	0	0.91229
Complement C2	CO2_MOUSE	85 kDa	0	1.0998	1.1343	1.2964	3.0414	0.91229
Ficolin-1	FCN1_MOUSE	36 kDa	1.9484	1.0998	2.2686	1.2964	3.0414	0.91229
Mannan-binding lectin serine protease 2	MASP2_MOUSE	76 kDa	0.97418	2.1997	0	0	3.0414	0.91229
Pigment epithelium-derived factor	PEDF_MOUSE	46 kDa	2.9225	1.0998	0	0	4.5621	0.91229

von Willebrand factor	VWF_MOUSE	309 kDa	0	0	2.2686	3.8893	2.281	0.91229
Kinesin family member 13B	A0A286YCV9_MOUSE	205 kDa	0	0	0	1.2964	0	0.91229
Anaphylatoxin-like domain-containing protein	A2AS37_MOUSE	40 kDa	0	1.0998	3.4029	3.8893	0.76034	0.91229
Peptidyl-prolyl cis-trans isomerase A	PPIA_MOUSE	18 kDa	1.9484	3.2995	3.4029	1.2964	0.76034	0.91229
Coagulation factor IX	FA9_MOUSE	53 kDa	0	1.0998	2.2686	2.5928	1.5207	0.91229
Complement component 7	D3YXF5_MOUSE	93 kDa	0	1.0998	1.1343	1.2964	3.0414	0.91229
Nestin	NEST_MOUSE	207 kDa	0.97418	1.0998	1.1343	0	0	0.91229
Tubulin beta-2A chain	TBB2A_MOUSE (+1)	50 kDa	0	0	0	0	0	0.91229
Tubulin beta-4A chain	TBB4A_MOUSE	50 kDa	0	0	0	0	0	0.91229
Tubulin beta-4B chain	TBB4B_MOUSE	50 kDa	0	0	1.1343	0	0	0.91229
Myosin-9	MYH9_MOUSE	226 kDa	0	0	0	0	0.76034	0.91229
Ig-like domain-containing protein	A0A0B4J1I5_MOUSE	13 kDa	0.97418	2.1997	1.1343	1.2964	0	0.91229
FAT atypical cadherin 1	A0A1L1SQU7_MOUSE	512 kDa	0	1.0998	0	0	0	0.91229
Heparanase	HPSE_MOUSE	60 kDa	0.97418	2.1997	2.2686	1.2964	0.76034	0.91229
Cluster of 14-3-3 protein zeta/delta	1433Z_MOUSE [4]	28 kDa	0	0	0	1.2964	0.76034	0.91229
14-3-3 protein zeta/delta	1433Z_MOUSE	28 kDa	0	0	0	1.2964	0.76034	0.91229
Nucleoprotein TPR	TPR_MOUSE	274 kDa	0	0	0	0	0	0.91229
ATP-dependent RNA helicase DHX30	DHX30_MOUSE	137 kDa	0	0	0	0	0	0.91229
Nuclear receptor subfamily 1 group D member 2	NR1D2_MOUSE	64 kDa	0.97418	1.0998	1.1343	1.2964	0.76034	0.91229
Plastin-3	PLST_MOUSE	71 kDa	0	0	0	0	0	0.91229
Ig-like domain-containing protein	A0A0B4J1J7_MOUSE	11 kDa	1.9484	2.1997	1.1343	2.5928	0	0.91229
Fibulin-1	FBLN1_MOUSE	78 kDa	0	0	1.1343	1.2964	0.76034	0.91229
Ig lambda-2 chain V region	LV2A_MOUSE	12 kDa	0.97418	1.0998	1.1343	1.2964	0	0.91229
Telomere-associated protein RIF1	RIF1_MOUSE	266 kDa	0	0	0	0	0.76034	0.91229
Triosephosphate isomerase	TPIS_MOUSE	32 kDa	0	1.0998	0	1.2964	0.76034	0.91229
G_PROTEIN_RECEP_F3_4 domain-containing protein	E9Q759_MOUSE	96 kDa	0	0	0	1.2964	0	0.91229
Neuron navigator 3	NAV3_MOUSE	252 kDa	0	0	0	0	0	0.91229
RRP12-like protein	RRP12_MOUSE	143 kDa	0	0	1.1343	0	0	0.91229
Sodium/potassium-transporting ATPase subunit alpha-2	AT1A2_MOUSE	112 kDa	0.97418	1.0998	0	0	0.76034	0.91229
M-phase phosphoprotein 9	MPP9_MOUSE	110 kDa	0	0	0	0	0	0.91229
WD repeat-containing protein 1	WDR1_MOUSE	66 kDa	0	0	0	0	0.76034	0.91229
Cluster of Rab GDP dissociation inhibitor beta	GDIB_MOUSE [2]	51 kDa	0	0	0	0	1.5207	0.91229
Rab GDP dissociation inhibitor beta	GDIB_MOUSE	51 kDa	0	0	0	0	1.5207	0.91229
Platelet glycoprotein V	GPV_MOUSE	63 kDa	0	0	0	0	0	0.91229
Ig-like domain-containing protein	A0A140T8M2_MOUSE	13 kDa	0.97418	2.1997	0	1.2964	0	0.91229
Transformation/transcription domain-associated protein	TRRAP_MOUSE	292 kDa	0	0	0	0	0	0.91229
Biotinidase	BTD_MOUSE	58 kDa	0	0	0	0	0	0.91229
Zinc finger protein 579	ZN579_MOUSE	61 kDa	0	0	0	0	0.76034	0.91229
L-selectin	LYAM1_MOUSE	42 kDa	0	0	0	0	0.76034	0.91229
Proteoglycan 4	PRG4_MOUSE	116 kDa	0	0	1.1343	1.2964	0.76034	0.91229
Kinesin-like protein KIF23	KIF23_MOUSE	109 kDa	0	1.0998	0	0	0.76034	0.91229
Arf-GAP with SH3 domain, ANK repeat and PH domain-containing protein 2	ASAP2_MOUSE	107 kDa	0	0	0	0	0	0.91229
Serine protease inhibitor A3B	SPA3B_MOUSE	47 kDa	0	0	0	0	0.76034	0.91229
Syncoilin	SYNCI_MOUSE	54 kDa	0	0	0	0	0	0.91229
Cluster of Ig kappa chain V-III region PC 2880/PC 1229	KV3A1_MOUSE [3]	12 kDa	0	0	0	1.2964	0	0.91229
Ig kappa chain V-III region PC 2880/PC 1229	KV3A1_MOUSE (+1)	12 kDa	0	0	0	1.2964	0	0.91229
Ig kappa chain V19-17	KV5A1_MOUSE	16 kDa	0	1.0998	1.1343	0	0	0.91229
Ig-like domain-containing protein	A0A075B5R3_MOUSE	13 kDa	0	0	0	0	0.76034	0.91229
InaD-like protein	INADL_MOUSE	199 kDa	0	0	0	0	0	0.91229
Ig-like domain-containing protein	A0A075B5N4_MOUSE	11 kDa	0	0	0	0	0	0.91229
Acetyl-CoA carboxylase 2	ACACB_MOUSE	276 kDa	0	0	1.1343	0	0	0.91229
Ig-like domain-containing protein	A0A075B5N6_MOUSE (+1)	13 kDa	0	1.0998	0	0	0	0.91229
Ig-like domain-containing protein	A0A075B603_MOUSE (+1)	12 kDa	0	0	0	0	0.76034	0.91229
Thyroxine-binding globulin	THBG_MOUSE	47 kDa	0	0	0	0	0.76034	0.91229
Ig-like domain-containing protein	A0A075B5K8_MOUSE (+2)	13 kDa	0	0	0	0	0	0.91229
Polyunsaturated fatty acid lipoxygenase ALOX12	LOX12_MOUSE	75 kDa	0	0	0	1.2964	0	0.91229
G_PROTEIN_RECEP_F1_2 domain-containing protein	Q9D3U9_MOUSE	35 kDa	0	0	0	0	0.76034	0.91229
Ig-like domain-containing protein	A0A075B5K9_MOUSE	12 kDa	0	0	0	0	0	0.91229
Ig-like domain-containing protein	A0A0B4J1I1_MOUSE	13 kDa	0	0	0	0	0	0.91229
Ig kappa chain V-VI region NQ2-6.1	KV6AB_MOUSE	12 kDa	0	0	0	0	0	0.91229
Peptidyl-prolyl cis-trans isomerase C	PPIC_MOUSE	23 kDa	0	0	0	0	0	0.91229
Ras-related protein Rab-18	RAB18_MOUSE	23 kDa	0	0	0	0	0	0.91229
Exosome complex component RRP4	EXOS2_MOUSE	33 kDa	0	0	0	0	0	0.91229
Activating signal cointegrator 1 complex subunit 2	ASCC2_MOUSE	86 kDa	0	0	0	0	0	0.91229
Alpha-2-macroglobulin-P	A2MG_MOUSE	164 kDa	0	0	0	0	0	0
Predicted gene 7298	A0A0N4SVU1_MOUSE	165 kDa	0	0	0	0	0	0
Ig-like domain-containing protein	Q4KN81_MOUSE	40 kDa	1.9484	0	5.6716	7.7785	0	0
Ig-like domain-containing protein	O19441_MOUSE	41 kDa	2.9225	0	0	0	0	0
H-2 class I histocompatibility antigen, Q7 alpha chain	HA17_MOUSE	38 kDa	0	0	0	5.1857	0	0
H-2 class I histocompatibility antigen, D-37 alpha chain	HA15_MOUSE	41 kDa	0	0	0	0	0	0
Keratin, type II cytoskeletal 6A	K2C6A_MOUSE	59 kDa	0	0	0	2.5928	0	0
IF rod domain-containing protein	A0A2R8VHP3_MOUSE	58 kDa	0	0	0	0	0	0
Keratin, type II cytoskeletal 75	K2C75_MOUSE	60 kDa	0	0	0	2.5928	0	0
Keratin, type II cytoskeletal 72	K2C72_MOUSE	57 kDa	0	0	0	0	0	0
IF rod domain-containing protein	Q6IFZ8_MOUSE	60 kDa	0	0	0	0	0	0
Keratin, type II cuticular Hb4	KRT84_MOUSE	65 kDa	0	0	0	0	0	0
Keratin, type II cytoskeletal 4	K2C4_MOUSE	56 kDa	0	0	0	0	0	0
Keratin, type II cytoskeletal 6B	K2C6B_MOUSE	60 kDa	0	0	0	0	0	0
Predicted gene 17087	V9GXQ2_MOUSE	18 kDa	0	0	0	0	0	0
Keratin, type I cytoskeletal 13	K1C13_MOUSE	48 kDa	0.97418	2.1997	1.1343	1.2964	0	0
Keratin, type I cytoskeletal 19	K1C19_MOUSE	45 kDa	0	1.0998	1.1343	2.5928	0	0
Keratin, type I cytoskeletal 27	K1C27_MOUSE	49 kDa	0	0	0	0	0	0
Keratin, type I cytoskeletal 25	K1C25_MOUSE	49 kDa	0	0	0	0	0	0
Epididymal secretory glutathione peroxidase	GPX5_MOUSE	25 kDa	0	0	0	2.5928	0	0
Predicted gene 8251	A0A571BEV2_MOUSE	747 kDa	0	1.0998	2.2686	0	1.5207	0
Titin	TITIN_MOUSE	3906 kDa	0	1.0998	0	0	0	0
Transcriptional regulator ATRX	ATRX_MOUSE	279 kDa	0.97418	0	0	0	0	0
Fibrinogen alpha chain	FIBA_MOUSE	87 kDa	1.9484	1.0998	7.9402	15.557	0	0
Properdin	PROP_MOUSE	50 kDa	5.8451	6.5991	6.8059	7.7785	0	0
CD5 antigen-like	CD5L_MOUSE	39 kDa	5.8451	6.5991	6.8059	7.7785	0	0
Maltase-glucoamylase 2, pseudogene	A0A571BDS5_MOUSE	264 kDa	0	0	0	0	0.76034	0
Cluster of Spectrin beta chain, erythrocytic	SPTB1_MOUSE [2]	245 kDa	0	0	11.343	5.1857	0	0
Spectrin beta chain, erythrocytic	SPTB1_MOUSE	245 kDa	0	0	11.343	5.1857	0	0
Spectrin beta chain, non-erythrocytic 1	SPTB2_MOUSE	274 kDa	0	0	2.2686	0	0	0
Spectrin alpha chain, erythrocytic 1	SPTA1_MOUSE	280 kDa	0	0	21.552	9.0749	0	0
PAX3- and PAX7-binding protein 1	PAXB1_MOUSE	105 kDa	5.8451	1.0998	1.1343	1.2964	0.76034	0
Olfactory receptor	Q8VFB1_MOUSE	35 kDa	0	0	3.4029	3.8893	0	0
Leukemia inhibitory factor receptor	LIFR_MOUSE	123 kDa	2.9225	1.0998	2.2686	0	6.0827	0
Band 3 anion transport protein	B3AT_MOUSE	103 kDa	0	0	20.418	2.5928	0.76034	0
Ankyrin-1	ANK1_MOUSE	204 kDa	0	0	15.88	6.4821	0	0
Immunoglobulin heavy constant alpha	A0A0A6YXW6_MOUSE	42 kDa	4.8709	4.3994	4.5373	2.5928	1.5207	0
Predicted gene 4788	E9Q8B5_MOUSE	99 kDa	1.9484	3.2995	7.9402	5.1857	2.281	0
SCY domain-containing protein	Q9EQI5_MOUSE	12 kDa	4.8709	6.5991	3.4029	7.7785	0	0
Dynein heavy chain 2, axonemal	DYH2_MOUSE	512 kDa	0	0	1.1343	0	0	0
Immunoglobulin J chain	IGJ_MOUSE	18 kDa	2.9225	4.3994	7.9402	6.4821	0	0
Filamin-A	FLNA_MOUSE	281 kDa	0	2.1997	3.4029	6.4821	0.76034	0
Selenoprotein P	SEPP1_MOUSE	43 kDa	1.9484	1.0998	0	0	3.0414	0

Cluster of Heat shock cognate 71 kDa protein	HSP7C_MOUSE [2]	71 kDa	0	1.0998	5.6716	3.8893	2.281	0
Heat shock cognate 71 kDa protein	HSP7C_MOUSE	71 kDa	0	1.0998	5.6716	3.8893	2.281	0
Heat shock-related 70 kDa protein 2	HSP72_MOUSE	70 kDa	0	0	3.4029	0	0	0
Fibrinogen beta chain	FIBB_MOUSE	55 kDa	0	0	5.6716	6.4821	0	0
Gp_dh_C domain-containing protein	V9GXA7_MOUSE	13 kDa	0	0	0	0	0	0
Fibrinogen gamma chain	FIBG_MOUSE	49 kDa	0	0	6.8059	12.964	0.76034	0
BRCA1-A complex subunit RAP80	UIMC1_MOUSE	81 kDa	1.9484	2.1997	0	0	0	0
Nucleotide exchange factor SIL1	SIL1_MOUSE	52 kDa	0	0	1.1343	0	0.76034	0
Alpha-synuclein	SYUA_MOUSE	14 kDa	0	0	10.209	6.4821	0	0
Lipocln_cytosolic_FA-bd_dom domain-containing protein	A2BIN1_MOUSE (+1)	21 kDa	3.8967	2.1997	2.2686	0	0	0
Ig-like domain-containing protein	A0A075B5P7_MOUSE	13 kDa	0	0	0	0	0	0
Ig heavy chain V region X24	HVM39_MOUSE	13 kDa	0	0	0	0	0	0
Ig heavy chain V region RF	HVM53_MOUSE	13 kDa	0	0	0	0	0	0
Nesprin-1	SYNE1_MOUSE	1010 kDa	0	0	1.1343	0	0	0
Adenomatous polyposis coli protein	APC_MOUSE	311 kDa	0.97418	0	1.1343	0	0	0
Lutropin-choriogonadotropic hormone receptor	LSHR_MOUSE	78 kDa	0	1.0998	2.2686	1.2964	0	0
Ig-like domain-containing protein	A0A075B5T3_MOUSE (+1)	13 kDa	0	0	0	0	0	0
Phosphatidate phosphatase LPIN1	LPIN1_MOUSE	102 kDa	0	0	1.1343	2.5928	0	0
Platelet glycoprotein Ib alpha chain	GP1BA_MOUSE	80 kDa	0.97418	2.1997	0	0	1.5207	0
Platelet factor 4	PLF4_MOUSE	11 kDa	0.97418	1.0998	1.1343	0	0	0
Dual specificity phosphatase DUPD1	DUPD1_MOUSE	24 kDa	4.8709	0	1.1343	0	0	0
Progranulin	GRN_MOUSE	63 kDa	2.9225	2.1997	3.4029	2.5928	0	0
Protein Daple	DAPLE_MOUSE	227 kDa	1.9484	0	1.1343	1.2964	0	0
Prolactin-2B1	PR2B1_MOUSE	26 kDa	0	1.0998	0	0	0	0
Angiotensinogen	ANGT_MOUSE	52 kDa	1.9484	1.0998	0	1.2964	0.76034	0
Microtubule-associated serine/threonine-protein kinase 4	MAST4_MOUSE	284 kDa	0	2.1997	1.1343	0	0	0
Hyaluronan-binding protein 2	HABP2_MOUSE	62 kDa	0	0	3.4029	3.8893	0	0
Transitional endoplasmic reticulum ATPase	TERA_MOUSE	89 kDa	0	1.0998	0	0	0.76034	0
Neurobeachin	NBEA_MOUSE	327 kDa	0	0	1.1343	0	0	0
Ig-like domain-containing protein	A0A075B677_MOUSE	10 kDa	5.8451	2.1997	3.4029	1.2964	0	0
Pericentrin	PCNT_MOUSE	329 kDa	0	0	0	0	0.76034	0
Polycystic kidney disease protein 1-like 1	PK1L1_MOUSE	291 kDa	0	1.0998	0	0	0	0
Ig-like domain-containing protein	A0A140T8P9_MOUSE (+2)	13 kDa	1.9484	1.0998	1.1343	5.1857	0	0
Keratin, type I cytoskeletal 28	K1C28_MOUSE	50 kDa	0.97418	5.4992	1.1343	3.8893	0	0
Rab-like protein 6	RABL6_MOUSE	80 kDa	0	0	1.1343	0	0	0
Dynein, axonemal, heavy chain 9	B1AR51_MOUSE	512 kDa	0.97418	0	0	0	0	0
Lipoxygenase homology domain-containing protein 1	LOXH1_MOUSE	236 kDa	0	1.0998	0	0	0	0
Mannose-binding protein A	MBL1_MOUSE	25 kDa	1.9484	1.0998	1.1343	1.2964	1.5207	0
Cytochrome P450 1A2	CP1A2_MOUSE	58 kDa	0.97418	0	1.1343	0	0.76034	0
Apolipoprotein C-II	APOC2_MOUSE	11 kDa	0	0	1.1343	1.2964	1.5207	0
Midasin	A2ANY6_MOUSE	630 kDa	0.97418	0	0	0	0.76034	0
Predicted gene 597	E9Q8J5_MOUSE	100 kDa	0	0	0	1.2964	0	0
Endoplasmic reticulum metallopeptidase 1	ERMP1_MOUSE	100 kDa	0	0	0	0	0.76034	0
Cluster of Alpha-amylase 1	AMY1_MOUSE [2]	58 kDa	0.97418	2.1997	0	1.2964	0	0
Alpha-amylase 1	AMY1_MOUSE	58 kDa	0.97418	2.1997	0	1.2964	0	0
Pancreatic alpha-amylase	AMYP_MOUSE	57 kDa	0	0	0	0	0	0
Cluster of Sal-like protein 1	SALL1_MOUSE [2]	140 kDa	0	0	0	0	0.76034	0
Sal-like protein 1	SALL1_MOUSE	140 kDa	0	0	0	0	0.76034	0
Sal-like protein 3	SALL3_MOUSE	139 kDa	0	0	0	0	0.76034	0
Extracellular matrix protein 1	ECM1_MOUSE	63 kDa	0	0	3.4029	1.2964	0	0
Cofilin-1	COF1_MOUSE	19 kDa	0	0	3.4029	2.5928	0.76034	0
Thioredoxin	THIO_MOUSE	12 kDa	0.97418	1.0998	3.4029	3.8893	0	0
Pyruvate kinase PKM	KPYM_MOUSE	58 kDa	0	2.1997	1.1343	0	0	0
Proliferation marker protein Ki-67	KI67_MOUSE	351 kDa	0.97418	0	0	0	0	0
E3 ubiquitin-protein ligase RNF146	RN146_MOUSE	39 kDa	0	0	0	0	0.76034	0
Coiled-coil domain-containing protein 150	CC150_MOUSE	129 kDa	0.97418	0	0	0	0	0
AP2-interacting clathrin-endocytosis protein	K1107_MOUSE	134 kDa	0	0	1.1343	0	0	0
Intraflagellar transport protein 172 homolog	IF172_MOUSE	198 kDa	0	0	1.1343	0	0	0
14-3-3 protein gamma	1433G_MOUSE	28 kDa	0	0	0	0	0	0
14-3-3 protein epsilon	1433E_MOUSE	29 kDa	0	0	0	0	0	0
14-3-3 protein theta	1433T_MOUSE	28 kDa	0	0	0	0	0	0
Cluster of Alpha-actinin-1	ACTN1_MOUSE [2]	103 kDa	0	0	0	0	1.5207	0
Alpha-actinin-1	ACTN1_MOUSE	103 kDa	0	0	0	0	1.5207	0
Alpha-actinin-4	ACTN4_MOUSE	105 kDa	0	0	0	0	1.5207	0
C-reactive protein	CRP_MOUSE	25 kDa	0.97418	2.1997	1.1343	1.2964	0	0
Histone-lysine N-methyltransferase 2D	KMT2D_MOUSE	600 kDa	0	0	1.1343	1.2964	0	0
Pleckstrin homology domain-containing family A member 6	PKHA6_MOUSE	131 kDa	0	0	0	1.2964	0.76034	0
Cluster of Protocadherin alpha 11	Q91Y19_MOUSE [5]	103 kDa	0	0	0	2.5928	0	0
Protocadherin alpha 11	Q91Y19_MOUSE	103 kDa	0	0	0	2.5928	0	0
Protocadherin alpha-10	PCDAA_MOUSE	102 kDa	0	0	0	2.5928	0	0
Protocadherin alpha-9	PCDA9_MOUSE	107 kDa	0	0	0	2.5928	0	0
Protocadherin alpha 5	Q91Y15_MOUSE	102 kDa	0	0	0	0	0	0
Protocadherin alpha-7	PCDA7_MOUSE	101 kDa	0	0	0	0	0	0
Ig-like domain-containing protein	A0A075B5S2_MOUSE	14 kDa	2.9225	1.0998	1.1343	1.2964	0	0
Ig-like domain-containing protein	A0A075B5R0_MOUSE	13 kDa	1.9484	3.2995	0	1.2964	0	0
Protein unc-45 homolog B	UN45B_MOUSE	104 kDa	0	0	0	0	0.76034	0
TRIO and F-actin-binding protein	TARA_MOUSE	223 kDa	0	0	0	0	0.76034	0
Disco-interacting protein 2 homolog B	DIP2B_MOUSE	171 kDa	0	0	0	0	0.76034	0
Ig-like domain-containing protein	A0A075B5J9_MOUSE (+1)	12 kDa	0.97418	2.1997	0	1.2964	0	0
Max-like protein X	MLX_MOUSE	33 kDa	0	0	0	1.2964	0	0
Fatty acid-binding protein, adipocyte	FABP4_MOUSE	15 kDa	0	0	1.1343	0	0	0
Protein turtle homolog A	TUTLA_MOUSE	127 kDa	0.97418	0	0	0	0	0
DNA topoisomerase 2-alpha	TOP2A_MOUSE	173 kDa	0	0	0	0	0.76034	0
C2 domain-containing protein	A0A286YDU8_MOUSE	162 kDa	0.97418	0	0	0	0	0
Tyrosine--tRNA ligase, mitochondrial	SYYM_MOUSE	53 kDa	0.97418	0	0	0	0	0
Endoplasmic reticulum chaperone BiP	BIP_MOUSE	72 kDa	0	0	3.4029	1.2964	0.76034	0
Ras-related C3 botulinum toxin substrate 1	RAC1_MOUSE	21 kDa	0	0	2.2686	1.2964	0	0
Ras-related protein Rap-1b	RAP1B_MOUSE	21 kDa	0	0	2.2686	3.8893	0	0
Myosin light polypeptide 6	MYL6_MOUSE	17 kDa	0	1.0998	2.2686	2.5928	0.76034	0
Complement C1q subcomponent subunit B	C1QB_MOUSE	27 kDa	0.97418	2.1997	1.1343	1.2964	0	0
Hormone-sensitive lipase	LIPS_MOUSE	83 kDa	0.97418	0	0	0	0.76034	0
Peptidase inhibitor 16	PI16_MOUSE	54 kDa	0.97418	0	1.1343	0	0	0
Adenylate cyclase type 3	ADCY3_MOUSE	129 kDa	0.97418	1.0998	0	0	0	0
Immunoglobulin superfamily containing leucine-rich repeat protein 2	ISLR2_MOUSE	80 kDa	0	1.0998	0	0	0	0
Vinculin	VINC_MOUSE	117 kDa	0	1.0998	1.1343	1.2964	0	0
Proprotein convertase subtilisin/kexin type 5	PCSK5_MOUSE	209 kDa	0	0	3.4029	1.2964	0	0
ELKS/Rab6-interacting/CAST family member 1	RB6I2_MOUSE	128 kDa	0.97418	0	0	0	0	0
Optineurin	OPTN_MOUSE	67 kDa	0	0	0	1.2964	0	0
Ropporin-1-like protein	ROP1L_MOUSE	25 kDa	0.97418	1.0998	0	0	0	0
Rab GDP dissociation inhibitor alpha	GDIA_MOUSE	51 kDa	0	0	0	0	0.76034	0
Cluster of Retinal dehydrogenase 1	AL1A1_MOUSE [2]	54 kDa	0	0	1.1343	0	1.5207	0
Retinal dehydrogenase 1	AL1A1_MOUSE	54 kDa	0	0	1.1343	0	1.5207	0
Aldehyde dehydrogenase, cytosolic 1	AL1A7_MOUSE	55 kDa	0	0	0	0	0	0
Fermitin family homolog 3	URP2_MOUSE	76 kDa	0	0	2.2686	1.2964	1.5207	0
Superoxide dismutase [Cu-Zn]	SODC_MOUSE	16 kDa	1.9484	1.0998	0	0	0	0
Peroxiredoxin-6	PRDX6_MOUSE	25 kDa	0	0	1.1343	1.2964	0	0
Suprabasin	SBSN_MOUSE	72 kDa	0	0	1.1343	0	0	0

Cystatin domain-containing protein	Q9D1B1_MOUSE	17 kDa	0	0	0	1.2964	0	0
Protein CTLA-2-alpha	CTL2A_MOUSE	16 kDa	0	0	1.1343	0	0	0
Ig-like domain-containing protein	A0A140T8P3_MOUSE	13 kDa	1.9484	1.0998	1.1343	1.2964	0	0
Ig-like domain-containing protein	A0A075B5T5_MOUSE	11 kDa	0	2.1997	0	3.8893	0	0
AT-rich interactive domain-containing protein 1A	ARI1A_MOUSE	242 kDa	0	1.0998	0	0	0.76034	0
Cholinesterase	CHLE_MOUSE	68 kDa	0	1.0998	0	0	0	0
Protein BHLHb9	BHLH9_MOUSE	60 kDa	0	0	0	1.2964	0	0
Dual specificity tyrosine-phosphorylation-regulated kinase 2	DYRK2_MOUSE	67 kDa	0	1.0998	0	0	0	0
Lysosomal-trafficking regulator	LYST_MOUSE	425 kDa	0	1.0998	0	0	0	0
G_PROTEIN_RECEP_F3_4 domain-containing protein	H3BK37_MOUSE	98 kDa	0.97418	0	0	0	0	0
Choline-phosphate cytidylyltransferase A	PCY1A_MOUSE	42 kDa	0	0	1.1343	1.2964	0	0
Filamin A-interacting protein 1-like	FIL1L_MOUSE	130 kDa	0	0	1.1343	0	0	0
Polymeric immunoglobulin receptor	PIGR_MOUSE	85 kDa	0	0	0	0	0.76034	0
Diacylglycerol kinase	D3YWQ0_MOUSE	118 kDa	0.97418	0	0	0	0	0
Adenylate cyclase type 10	ADCYA_MOUSE	186 kDa	0	0	1.1343	0	0	0
Cluster of Fructose-bisphosphate aldolase	A6ZI47_MOUSE [2]	39 kDa	0	0	1.1343	1.2964	0	0
Fructose-bisphosphate aldolase	A6ZI47_MOUSE	39 kDa	0	0	1.1343	1.2964	0	0
Fructose-bisphosphate aldolase A	ALDOA_MOUSE	39 kDa	0	0	1.1343	1.2964	0	0
Cluster of GTP-binding nuclear protein Ran	RAN_MOUSE [2]	24 kDa	0	0	1.1343	0	0	0
GTP-binding nuclear protein Ran	RAN_MOUSE	24 kDa	0	0	1.1343	0	0	0
GTP-binding nuclear protein Ran	Q14AA6_MOUSE	24 kDa	0	0	1.1343	0	0	0
Cathelicidin antimicrobial peptide	CAMP_MOUSE	19 kDa	0	0	2.2686	1.2964	0	0
Complement C1q subcomponent subunit C	C1QC_MOUSE	26 kDa	0	0	2.2686	1.2964	0	0
Periostin	POSTN_MOUSE	93 kDa	0	0	2.2686	0	0	0
Protein 4.1	41_MOUSE	96 kDa	0	0	2.2686	1.2964	0	0
Protein RUFY3	RUFY3_MOUSE	53 kDa	0	0	0	0	0.76034	0
Nucleosome assembly protein 1-like 3	NP1L3_MOUSE	61 kDa	0	0	0	1.2964	0	0
NAC-alpha domain-containing protein 1	NACAD_MOUSE	157 kDa	0	0	0	1.2964	0	0
Glutathione peroxidase 1	GPX1_MOUSE	22 kDa	0	0	1.1343	1.2964	0.76034	0
Transgelin-2	TAGL2_MOUSE	22 kDa	0	0	1.1343	1.2964	0	0
Breast cancer anti-estrogen resistance protein 1	BCAR1_MOUSE	94 kDa	0	0	0	0	0.76034	0
Myosin-binding protein C, slow-type	A0A571BEN1_MOUSE	128 kDa	0	0	0	1.2964	0	0
Proprotein convertase subtilisin/kexin type 7	PCSK7_MOUSE	84 kDa	0.97418	0	0	0	0	0
Lipid droplet-associated hydrolase	LDAH_MOUSE	37 kDa	0	1.0998	0	0	0	0
Disintegrin and metalloproteinase domain-containing protein 28	ADA28_MOUSE	89 kDa	0	0	1.1343	1.2964	0	0
TGF-beta-activated kinase 1 and MAP3K7-binding protein 2	TAB2_MOUSE	76 kDa	0.97418	0	0	0	0	0
Zinc finger CCCH type-containing 7 A	E9PWW6_MOUSE	111 kDa	0	0	1.1343	0	0	0
Myosin-7B	MYH7B_MOUSE	222 kDa	0	0	0	0	0.76034	0
Hepatocyte growth factor receptor	MET_MOUSE	154 kDa	0	0	0	0	0.76034	0
Ig-like domain-containing protein	A0A075B5P0_MOUSE	13 kDa	0	0	0	0	0	0
Cluster of Ig-like domain-containing protein	A0A075B5S9_MOUSE [2]	11 kDa	0.97418	1.0998	0	0	0	0
Ig-like domain-containing protein	A0A075B5S9_MOUSE	11 kDa	0.97418	1.0998	0	0	0	0
Ig-like domain-containing protein	A0A0B4J1J4_MOUSE	13 kDa	0.97418	1.0998	0	0	0	0
EGF-containing fibulin-like extracellular matrix protein 1	FBLN3_MOUSE	55 kDa	0	0	3.4029	0	0	0
Creatine kinase M-type	KCRM_MOUSE	43 kDa	0	0	2.2686	0	0	0
Ig-like domain-containing protein	A0A075B5Y1_MOUSE (+1)	13 kDa	0.97418	1.0998	0	0	0	0
Ran-specific GTPase-activating protein	RANG_MOUSE	24 kDa	0	0	1.1343	0	0	0
Ig-like domain-containing protein	A0A075B5M1_MOUSE	10 kDa	0	1.0998	0	2.5928	0	0
Ig-like domain-containing protein	A0A075B5U1_MOUSE	11 kDa	0	1.0998	0	1.2964	0	0
Ig-like domain-containing protein	A0A140T8M6_MOUSE (+1)	13 kDa	0	1.0998	1.1343	1.2964	0	0
Histidine triad nucleotide-binding protein 1	HINT1_MOUSE	14 kDa	0	0	1.1343	1.2964	0	0
55 kDa erythrocyte membrane protein	EM55_MOUSE	52 kDa	0	0	1.1343	0	0.76034	0
Coagulation factor XIII A chain	F13A_MOUSE	83 kDa	0	0	0	0	0.76034	0
Lactoylglutathione lyase	LGUL_MOUSE	21 kDa	0	0	0	0	0.76034	0
Cytochrome c, somatic	CYC_MOUSE	12 kDa	0.97418	0	0	0	0	0
Bisphosphoglycerate mutase	PMGE_MOUSE	30 kDa	0.97418	0	0	0	1.5207	0
Placenta-specific protein 9	PLAC9_MOUSE	11 kDa	0	1.0998	0	0	0	0
IRG-type G domain-containing protein	Q3UED7_MOUSE	47 kDa	0	0	0	0	0.76034	0
Nucleolar RNA helicase 2	DDX21_MOUSE	94 kDa	0.97418	0	0	0	0	0
Keratin, type I cytoskeletal 26	K1C26_MOUSE	51 kDa	0	0	0	1.2964	0	0
G_PROTEIN_RECEP_F3_4 domain-containing protein	E9Q2U5_MOUSE	98 kDa	0	0	0	0	1.5207	0
Profilin-1	PROF1_MOUSE	15 kDa	0	1.0998	1.1343	1.2964	0	0
Ubiquitin-like modifier-activating enzyme 1	UBA1_MOUSE	118 kDa	0	0	0	0	0.76034	0
Exosome complex exonuclease RRP44	RRP44_MOUSE	109 kDa	0	1.0998	0	0	0	0
Translocation protein SEC63 homolog	SEC63_MOUSE	88 kDa	0	1.0998	0	0	0	0
Centromere protein L	CENPL_MOUSE	38 kDa	0	0	0	1.2964	0	0
Microtubule-associated protein 2	MTAP2_MOUSE	199 kDa	0	0	0	1.2964	0	0
Collagen alpha-1(XI) chain	COBA1_MOUSE	181 kDa	0	0	1.1343	0	0	0
Ig-like domain-containing protein	A0A075B5Q6_MOUSE	13 kDa	0	2.1997	0	0	0	0
Basigin	BASI_MOUSE	42 kDa	0	0	2.2686	0	0	0
Erythrocyte band 7 integral membrane protein	STOM_MOUSE	31 kDa	0	0	1.1343	0	0	0
Ig-like domain-containing protein	A0A075B697_MOUSE (+2)	12 kDa	0	1.0998	0	0	0	0
Thrombospondin-4	TSP4_MOUSE	106 kDa	0	0	2.2686	0	0	0
Complement C1q subcomponent subunit A	C1QA_MOUSE	26 kDa	0.97418	0	0	0	0.76034	0
Ig-like domain-containing protein	A0A075B5N9_MOUSE (+1)	11 kDa	0	1.0998	0	1.2964	0	0
Protein sel-1 homolog 2	SE1L2_MOUSE	78 kDa	0.97418	0	0	0	0	0
Nucleobindin-1	NUCB1_MOUSE	53 kDa	0	0	1.1343	0	0	0
Transcription factor SOX-9	SOX9_MOUSE	56 kDa	0	0	0	0	0.76034	0
Zinc finger protein 418	Q8BFS8_MOUSE	75 kDa	0	0	1.1343	0	0	0
Protein SFI1 homolog	SFI1_MOUSE	144 kDa	0.97418	0	0	0	0	0
Liprin-alpha-3	LIPA3_MOUSE	133 kDa	0	1.0998	0	0	0	0
Cadherin-5	CADH5_MOUSE	88 kDa	0	0	1.1343	0	0	0
PDZ and LIM domain protein 1	PDLI1_MOUSE	36 kDa	0	0	0	1.2964	0	0
Unconventional myosin-XIX	MYO19_MOUSE	108 kDa	0	0	1.1343	0	0	0
Ig-like domain-containing protein	A0A0A6YXQ0_MOUSE	13 kDa	0	1.0998	0	0	0	0
Predicted gene 13084	A2A8N0_MOUSE (+1)	57 kDa	0	1.0998	0	0	0	0
Ig heavy chain V region	HVM00_MOUSE	13 kDa	0	1.0998	0	0	0	0
Ig kappa chain V-V region MOPC 41	KV5A7_MOUSE	14 kDa	0	1.0998	0	0	0	0
Apolipoprotein A-V	APOA5_MOUSE	41 kDa	0	0	0	1.2964	0	0
Glycosylation-dependent cell adhesion molecule 1	GLCM1_MOUSE	16 kDa	0	0	1.1343	0	0	0
tRNA N(3)-methylcytidine methyltransferase METTL6	METL6_MOUSE	33 kDa	0	0	1.1343	0	0	0
Monocarboxylate transporter 1	MOT1_MOUSE	53 kDa	0	0	1.1343	0	0	0
Proteasome subunit alpha type-6	PSA6_MOUSE	27 kDa	0	0	0	0	0.76034	0
BTB domain-containing protein	B8JKF6_MOUSE	58 kDa	0	1.0998	0	0	0	0
Plastin-2	PLSL_MOUSE	70 kDa	0	0	0	0	0.76034	0
Calponin-2	CNN2_MOUSE	33 kDa	0	0	0	1.2964	0	0
Proteasome subunit beta type-2	PSB2_MOUSE	23 kDa	0	0	0	0	0.76034	0
Metalloproteinase inhibitor 3	TIMP3_MOUSE	24 kDa	0	0	0	0	0.76034	0
Submaxillary gland androgen-regulated protein 3A	SMR3A_MOUSE	16 kDa	0	0	0	1.2964	0	0
Coenzyme Q-binding protein COQ10 homolog B, mitochondrial	CQ10B_MOUSE	27 kDa	0	0	0	1.2964	0	0
Armadillo repeat-containing X-linked protein 3	ARMX3_MOUSE	43 kDa	0	0	0	0	0.76034	0
Ig-like domain-containing protein	A0A075B5V8_MOUSE	11 kDa	0	0	1.1343	0	0	0
T-complex protein 1 subunit zeta	TCPZ_MOUSE	58 kDa	0	1.0998	0	0	0	0
NACHT, LRR and PYD domains-containing protein 5	NALP5_MOUSE	131 kDa	0	0	1.1343	0	0	0
